# NbCycB2 represses Nbwo activity via a negative feedback loop in tobacco trichome development

**DOI:** 10.1093/jxb/erz542

**Published:** 2020-01-28

**Authors:** Min-Liang Wu, Yu-Chao Cui, Li Ge, Li-Peng Cui, Zhi-Chao Xu, Hong-Ying Zhang, Zhao-Jun Wang, Dan Zhou, Shuang Wu, Liang Chen, Hong Cui

**Affiliations:** 1 Xiamen Key Laboratory for Plant Genetics, School of Life Sciences, Xiamen University, Xiamen, China; 2 Key Laboratory for Cultivation of Tobacco Industry, College of Tobacco Science, Henan Agricultural University, Zhengzhou, China; 3 FAFU-UCR Joint Center and Fujian Provincial Key Laboratory of Haixia Applied Plant Systems Biology, College of Horticulture, Fujian Agriculture and Forestry University, Fuzhou, China; 4 CNRS/Ecole Normale Superieure de Lyon, France

**Keywords:** Feedback loop, L1-like box, NbCycB2, Nbwo, *Nicotiana benthamiana*, trichome formation, woolly motif

## Abstract

The transcription factor *Woolly* (*Wo*) and its downstream gene *CycB2* have been shown to regulate trichome development in tomato (*Solanum lycopersicum*). It has been demonstrated that only the gain-of-function allele of *Slwo* (*SlWo*^*V*^, the *Slwo* woolly motif mutant allele) can increase the trichome density; however, it remains unclear why the two alleles function differently in trichome development. In this study, we used *Nicotiana benthamiana* as a model and cloned the homologues of *Slwo* and *SlCycB2* (named *Nbwo* and *NbCycB2*). We also constructed a *Nbwo* gain-of-function allele with the same mutation site as *SlWo*^*V*^ (named *NbWo*^*V*^). We found that both Nbwo and NbWo^V^ directly regulate *NbCycB2* and their own expression by binding to the promoter of *NbCycB2* and their own genomic sequences. As form of a feedback regulation, NbCycB2 negatively regulates trichome formation by repressing Nbwo activity at the protein level. We also found that mutations in the Nbwo woolly motif can prevent repression of NbWo^V^ by NbCycB2, which results in a significant increase in the amount of active Nbwo proteins and in increases in trichome density and the number of branches. Our results reveal a novel reciprocal regulation mechanism between *NbCycB2* and *Nbwo* during trichome formation in *N. benthamiana*.

## Teaser

*NbCycB2* is specifically expressed in trichomes of *Nicotiana benthamiana* and inhibits their formation by repressing Nbwo activity via a negative feedback loop.

## Introduction

Trichomes are specialized epidermal protuberances that are found on aerial parts of nearly all terrestrial plants. They can be classified into different types according to cell numbers and shapes, namely unicellular/multicellular, and glandular/non-glandular. It has been demonstrated that the development of trichomes in Arabidopsis (which are unicellular and non-glandular) is regulated by the trimeric MYB-bHLH-WDR complex of protein activators GL1-GL3/EGL-TTG13 (Oppenheimer *et al.*, 1991; Walker *et al.*, 1999; Payne *et al.*, 2000). This transcriptional complex activates the expression of the homeodomain protein GLABROUS2 (GL2) to induce the formation of trichomes (Rerie *et al.*, 1994; Grebe, 2012). It also triggers the expression of single-repeat R3 MYBs including TRY (Schnittger *et al.*, 1999), CPC (Wada *et al.*, 1997), ETC1, ETC2, ETC3 (Kirik *et al.*, 2004, Wester *et al.*, 2009), and TCL2 (Gan *et al.*, 2011), and these act as negative regulators of GL3 or EGL3 in trichome development by forming a repressor complex, GL3/EGL3-TRY/CPC-TTG1 (Wang *et al.*, 2008; Wester *et al.*, 2009). Thus, the control of trichome development in Arabidopsis requires a regulatory loop that includes both activators and repressors (Grebe, 2012; Pattanaik *et al.*, 2014).

Multicellular glandular secreting trichomes (GSTs) are present in ~30% of all vascular plants (Glas *et al.*, 2012). Since many useful phytochemical compounds are synthesized and secreted by such GSTs (Mauricio and Rausher, 1997; Hollósy, 2002; Valkama *et al.*, 2003; Freeman and Beattie, 2008), they have considerable potential economic potential (Sallets *et al.*, 2014; Huchelmann *et al.*, 2017). However, it has been shown that the networks that regulate unicellular trichomes do not function in the development of multicellular trichomes (Serna and Martin, 2006; Yang *et al.*, 2011; Kang *et al.*, 2016; Yan *et al.*, 2017).

In tomato (*Solanum lycopersicum*), a HD-ZIP IV transcription factor, Slwo, has been shown to regulate trichome initiation (Yang *et al.*, 2011). This contains four conserved domains, namely a homeodomain domain (HD), a leucine zipper (LZ) domain, a steroidogenic acute regulatory protein-related lipid transfer (START) domain, and a START-adjacent domain (SAD). However, overexpression of *Slwo* fails to induce a change of trichome density, and only ectopic expression of its gain-of-function mutant allele, *SlWo*^*V*^, can cause a higher density of trichomes in tomato and tobacco (*Nicotiana tabacum*) (Yang *et al.*, 2011, 2015). The *SlWo*^*V*^ allele has two point-mutations at the C-terminal domain (since this motif is conserved in most *Slwo* homologous genes, we name it as the ‘woolly motif’ in this study). Sequence analysis in Arabidopsis has shown that the Slwo protein is more similar to PROTODERMAL FACTOR2 (PDF2) and the PDF2 redundant protein MERISTEM L1 (ML1), both of which are involved in the differentiation of shoot epidermal cells (Abe *et al.*, 2001, Ogawa *et al.*, 2015), than to GL2.

The ectopic expression of a constitutive active B-type cyclin in Arabidopsis induces mitotic divisions and results in an increase in the number of multicellular trichomes (Schnittger *et al.*, 2005). SlCycB2, a hypothetical B-type cyclin, has been reported to directly interact with Slwo to promote the development of type I trichomes (Yang *et al.*, 2011, 2015). Its homologous protein in Arabidopsis (AtGIR1, AT5G06270) has also been found to interact with GL2 and co-repressor TOPLESS proteins (Wu and Citovsky, 2017*a*, 2017*b*). However, overexpression of *SlCycB2* results in a non-trichome phenotype, while suppression of *SlCycB2* promotes trichomes formation in tomato (Gao *et al.*, 2017). These inconsistent results raise important questions about the function of *SlCycB2* in trichome formation and why the mutation of the woolly motif can promote formation.

Similar to tomato, trichomes in *Nicotiana benthamiana* are typically multicellular structures, and almost all of them are glandular (Supplementary Fig. S1 at *JXB* online), making it a better system for their study than tomato. In addition, the genome map of *N. benthamiana* has been constructed (Bombarely *et al.*, 2012), and thus it represents an excellent model to study the molecular mechanisms of multicellular trichome formation (Goodin *et al.*, 2008). Here, we cloned the homologues of *Slwo* and *SlCycB2* in *N. benthamiana* (named *Nbwo* and *NbCycB2*, respectively) and constructed a previously identified two-point mutation of the *Nbwo* allele in the woolly motif, *NbWo*^*V*^ (Yang *et al.*, 2015). To investigate their biological functions in trichome development, we constructed transgenic lines with over- and underexpression of the genes. Our results demonstrate that Nbwo and NbWo^V^ can positively regulate the expression of *NbCycB2* through targeting to the *cis*-element in the promoter sequence. In contrast, NbCycB2 can act as a negative regulator of multicellular trichome formation by directly binding to and inhibiting the activity of Nbwo. The mutation of woolly motif blocked the interaction between NbCycB2 and Nbwo, thus removing the repression of Nbwo by NbCycB2 and resulting in increased trichome density. Our results reveal the mechanisms of the interaction between *Nbwo* and *NbCycB2* in regulating the development of glandular trichomes.

## Materials and methods

### Plant materials and growth conditions

Sterilized seeds of *Nicotiana benthamiana* were germinated and grown to seedlings under a photoperiod of 14/10 h light/dark (120 μmol m^–2^ s^–1^) at 26 °C on MS medium that was solidified with 0.8% (w/v) gellan gum. At 2 weeks old the plants were transferred to either sterilized bottles (for genetic transformation) or to soil in pots to grow to maturity. All wild-type and transgenic plants were grown in a greenhouse under a photoperiod of 14/10 h light/dark (120 μmol m^–2^ s^–1^) at 26 °C.

### Sequence analysis

The sequences of the similar proteins *Slwo* and *SlCycB2* were downloaded from the NCBI database (http://www.ncbi.nlm.nih.gov/) and the Sol Genomics Network (https://solgenomics.net/;Fernandez-Pozo *et al.*, 2015). Details of these proteins are given in Supplementary Tables S1 and S2. The aligned sequences were used to construct phylogenetic trees in MEGA 5 by using the maximum-likelihood (ML) criterion with 100 bootstraps. In addition, the relative conservation for each amino acid position in the protein sequences of Nbwo and NbCycB2 were evaluated using WebLoGo (https://weblogo.berkeley.edu/;Crooks *et al.*, 2004), followed by predictions of their conserved domains using SMART (https://smart.embl.de;Letunic and Bork, 2018).

### RNA extraction and real-time PCR

Total RNA was extracted from leaves of ~3-week-old plants by using an Eastep^®^ Super Total RNA Extraction Kit (Promega). The cDNA was synthesized using a M-MLV 1st Strand Kit (Invitrogen). Quantitative real-time PCR (qRT-PCR) was carried out using SYBR Premix Ex Taq II (TaKaRa). L25 ribosomal protein (L18908) was used as an endogenous control (Schmidt and Delaney, 2010). Primers are listed in Supplementary Table S3.

### Plasmid construction and transformation of *N. benthamiana*

The full-length coding sequences of *Nbwo* and *NbCycB2* were amplified from the general cDNA of *N. benthamiana* leaves. The *NbWo*^*V*^ allele with two point-mutations at loci 2084 (T replaced with G) and 2092 (G replaced with T) of *Nbwo* was generated by using a KOD -Plus- Mutagenesis Kit (Toyobo). To construct the overexpression lines of *Nbwo*, *NbWo*^*V*,^ and *NbCycB2*, these genes were inserted into the pCXSN-HA (*Nbwo* and *NbWo*^*V*^ fused to the HA tag) and pCXSN-FLAG (*NbCycB2* fused to the Flag tag) vectors under the control of the CaMV 35S promoter (Chen *et al.*, 2009). The underexpression vectors of *Nbwo* and *NbCycB2* were constructed by recombination with the RNAi vector pH7GWIWGII with the LR Clonase II enzyme (Invitrogen). Approximately 2800 bp of the upstream promoter sequences of *NbCycB2* and *Nbwo* were inserted into the pH2GW7 vector to create the promoter-driven GFP-GUS constructs (Cui *et al.*, 2015).

All of these constructs were transferred into *Agrobacterium tumefaciens* strain GV3101 to generate transgenic lines via *Agrobacterium*-mediated transformation. All the primers used are listed in Supplementary Table S3.

### Analysis of subcellular localization and tissue distribution

To analyse the subcellular localization of *Nbwo* and *NbWo*^*V*^, these genes were fused to green fluorescent protein (GFP) driven by the CaMV 35S promoter (*p35S::GFP-Nbwo*, *p35S::GFP-NbWo*^*V*^). The constructs were transferred into *A. tumefaciens* strain GV3101 and then transiently transformed into leaves of 4-week-old *N. benthamiana*. After cultivation under low light conditions for 48–72 h, GFP was observed using confocal microscopy (LSM 780, Carl Zeiss) with staining in DAPI solution (1 mg ml^–1^) for 15 min before observation. The subcellular localization of *NbCycB2* was observed in leaves of *NbCycB2*-overexpressing (-OE) transgenic plants (*p35S::GFP*-*NbCycB2*).

Tissue distribution assays were performed as described previously by Jefferson *et al.* (1987). GUS staining was repeated in at least three independent transgenic lines.

### Yeast hybrid assays

For yeast one-hybrid (Y1H) assays, the promoter of *NbCycB2*, separated into five fragments (E, –1027 to –831 bp; D, –830 to –631 bp; C, –630 to –411 bp; B, –410 to –201 bp; A, –200 to –1 bp), was amplified and inserted into the pHIS 2 vector (*NbCycB2*proE, *NbCycB2*proD, *NbCycB2*proC, *NbCycB2*proB, *NbCycB2*proA). Further investigations of the targeted sequences in the *NbCycB2* promoter were conducted by point-mutations in the two L1-like boxes in the D fragment: proD-m1, mutant one L1-like box, with 5´-GCAAATATTTACTC-3´ changed to 5´-GCGGGTGACTC-3´; and proD-m2, mutant two L1-like boxes, with 5´-GCAAATATTTACTC-3´ to 5´-GCGGGTGACTC-3´, and 5´-ATTTACTC-3´ changed to 5´-GGGACTCC-3´. To test the specific region of the *Nbwo* genomic sequence that binds with the Nbwo protein, four genomic fragments of *Nbwo* (G1, –8 to 251 bp including the T3 fragment; G2, 2169 to 2522 bp including the T4 fragment; G3, 3485 to 3780 bp including the T5 fragment; G4, 4333 to 4660 bp including the T6 fragment) were amplified and inserted into the pHIS 2 vector (*Nbwo-G1*, *Nbwo-G2*, *Nbwo-G3*, *Nbwo-G4*). The coding sequences (CDSs) of *Nbwo* and *NbWo*^*V*^ were fused to the GAL4 activation domain in pGADT7 vectors (AD-*Nbwo* and AD-*NbWo*^*V*^). The bait and prey constructs were co-transformed into *Saccharomyces cerevisiae* Y187 to test the DNA–protein interactions. The empty pGADT7 vector (AD) served as the negative control, and was cultivated on SD/–Leu/–Trp (–L–W) medium and tested on SD/–Leu/–His/–Trp (–L–-W–--H) medium with 60 mM 3-amino-1,2,4-triazole (Sangon Biotech Co., Ltd).

For yeast two-hybrid (Y2H) assays, four truncated *Nbwo* segments (including the HD, LZ domain, START domain, and SAD) and *NbCycB2* were fused to the GAL4 binding domain (BD-*Nbwo*-HD, BD-*Nbwo*-LZ, BD-*Nbwo*-START, BD-*Nbwo*-SAD, BD-*NbCycB2*) to verify the interactions with Nbwo or NbWo^V^. The *Nbwo* and *NbWo*^*V*^ genes fused to the GAL4 binding domain were used to test for auto-activation. Each pair of AD and BD plasmids were co-transformed into the Y2HGold yeast strain (Clontech). Clones containing the BD-53 and AD-T vectors served as positive controls, and BD-Lam and AD-T served as negative controls. The transformants were then cultivated on SD/–Leu/–Trp medium (DDO) and tested on SD/–Ade/–Leu/–His/–Trp medium with 40 mg l^–1^ X-a-Gal and 400 µg l^–1^ aureobasidin A (QDO/X/A).

For yeast three-hybrid (Y3H) assays, *Nbwo*-LZ was fused to the pBridge GAL4 binding domain, and *NbCycB2* was inserted downstream of the pBridge vector methionine repressible promoter (*Nbwo*-LZ+*NbCycB2*). The *BD-Nbwo-LZ* plasmid was transferred with AD-*Nbwo* as a positive control, and the empty pBridge vector was transferred with AD-*Nbwo* into Y2HGold strain as a negative control. The transformants were then tested on SD/–Ade/–Leu/–His/–Trp media with different concentrations of methionine (0 μM, 250 μM).

### Bimolecular fluorescence complementation assays

To examine the interactions of NbCycB2 and Nbwo (or NbWo^V^) in *N. benthamiana* protoplasts bimolecular fluorescence complementation (BiFC) assays were performed. The protoplasm was extracted from the leaves according to the method described by Yoo *et al.* (2007). The CDSs of *Nbwo*, *NbWo*^*V*^, and *NbCycB2* were each inserted into the pSAT6-cEYFP-C1-B vector (*2x35S::YFP*^*c*^*-Nbwo*, *2x35S::YFP*^*c*^*-NbWo*^*V*^) and the pSAT6-n(1–174)EYFP-C1 vector (*2x35S::YFP*^*n*^*-NbCycB2*) (Citovsky *et al.*, 2006). Pairs of the two plasmids were then transiently transformed into the protoplasts using the PEG–calcium transfection method as described by Yoo *et al.* (2007).

To determine the interactions between the Nbwo LZ domain and Nbwo itself, the CDSs of *Nbwo*, *NbWo*^*V*^, and *NbCycB2* were each fused to the C-terminal fragment of YFP in the p2YC vector; *Nbwo*-LZ and *NbWo*^*V*^ were also fused to the N-terminal fragment of YFP in the p2YN vector. Different plasmid combinations were co-infiltrated into leaves of *N. benthamiana* as described by (Shen *et al.*, 2011).

Fluorescence of the yellow fluorescent protein (YFP) was observed using confocal microscopy (LSM 780, Carl Zeiss). Three biological replicates were observed independently for each sample.

### Dual-luciferase assays

The regulatory effectors of *2x35S::HA-Nbwo*, *2x35s::HA-NbWo*^*V*^, and *2x35s::Flag-NbCycB2* were generated. The firefly luciferase reporters were driven by the B or D fragments of the *NbCycB2* promoter, and the *Renilla* luciferase was driven by the 35S promoter in the pGreen-0800-II report vector (*35S::REN-proB::LUC*, *35S::REN-proD::LUC*). Mutants of the *NbCycB2* promoter D fragments were also constructed using a mutagenesis kit (*35S::REN-proD-m2::LUC*; KOD -Plus- Mutagenesis Kit, Toyobo). The regulatory effector and reporter were used at a ratio of 5:1 or 5:5:1 for the expression tests of two or three plasmids.

### Immunoblotting and pull-down assays

Leaves of ~4-week-old *N. benthamiana* (~0.5 g) were homogenized in liquid nitrogen and then solubilized in 0.4 ml of lysis buffer (25 mM Tris-HCl, 2.5 mM EDTA, pH 8.0, 0.05% v/v NP-40, 5% glycerol, 150 mM NaCl, 1 mM phenylmethylsulphonyl fluoride, 20 µM MG132) for 30 min at 4 °C. Total protein (~80 µg) was then used for immunoblotting assays. After SDS-PAGE separation, the proteins were electrophoretically transferred to a PVDF membrane for immunodetection.

For pull-down assays of the interaction of NbCycB2 with Nbwo and NbWo^V^, the CDSs of *Nbwo*, *NbWo*^*V*^, and *NbCycB2* were inserted into the pET22b and PGEX-4T-1 vectors to create the fusion proteins (His-Nbwo, His-NbWo^V^, and GST-NbCycB2), and were then transformed into the *Escherichia coli* BL21 strain. The purified recombinant bait proteins (2 mg His-Nbwo or His-NbWo^V^) and prey proteins (2 mg GST-NbCycB2) were then mixed with 1 ml of binding buffer (50 mM Tris-HCl, pH 7.5, 0.6% Triton and X-100, 100 mM NaCl). After incubation at 4 °C for 2 h, 50 µl of glutathione agarose was added to the mixtures, followed by incubation for an additional 1 h. The immunoprecipitates were washed five times with binding buffer. The isolated proteins were detected by immunoblotting with anti-His or anti-GST antibodies.

The interaction of the NbCycB2 and Nbwo dimers was also determined by pull-down assays. *NbCycB2* was fused to the MBP tag and transformed into the *E. coli* BL21 strain. The MBP and NbCycB2-MBP proteins were purified using amylose resin (NEB). The *Nbwo* overexpression plasmids (*35S::HA-Nbwo* and *35S::Flag-Nbwo*) were transformed into *N. benthamiana* protoplasts according to the method described by Yoo *et al.* (2007). After transformation for 12 h, the HA-Nbwo and Flag-Nbwo proteins were each extracted from the protoplasts. Total protein was then uniformly mixed and incubated with purified MBP (2 mg) or NbCycB2-MBP (2 mg or 6 mg) proteins. Next, 5 µl of anti-HA antibody (Sigma) was added to each reaction tube. After incubation at 4 °C for 3 h, 10 µl of protein A magnetic beads (ThermoFisher Scientific) was added to the mixtures and they were further incubated for 1 h. The immunoprecipitates were washed five times with lysis buffer. The isolated proteins were detected by immunoblotting with anti-Flag or anti-MBP antibodies.

### Chromatin immunoprecipitation assays

*NbWo* ^*V*^-OE plants at 4 weeks old were used for the chromatin immunoprecipitation (ChIP) assay as described previously by (Gendrel *et al.*, 2005). The HA-NbWo^V^ proteins were precipitated using anti-HA antibody (Santa Cruz Biotechnology). Primers were designed to amplify three fragments (length ~120–210 bp) within the 1.7-kbp upstream sequence of the *NbCycB2* transcription start site, and seven fragments within ~8.7 kbp of the genomic DNA sequence of *Nbwo*. After immunoprecipitation, the purified DNA was analysed using real-time PCR. Enrichment was calculated from the ratio of the immunoprecipitated sequences.

### Observation of phenotypes

Leaves from 10-d-old plants of the transgenic lines and wild-type were fixed with 2% glutaraldehyde (0.1M phosphate buffer, PH 7.4) at 4 °C for 12 h for SEM analysis. The samples were then dehydrated with an ethanol series of 10–100% in steps of 10% for 20 min each time. Finally, the samples were dried in a critical-point drying device (Leica EMCPD030) and coated with gold particles before being observed using a JSM-6390/LV SEM.

### Accession numbers

The accession numbers of sequences reported here are as follows: *Nbwo* (Niben101Scf07790g01007.1), *Nbwo*-allele (Niben101 Scf00176g11005.1), *NbCycB2* (Niben101Scf10299g00003.1), *NbCycB2-* allele (Niben101Scf10396g00002.1), *NbML1* (Niben101Scf00703g 00003.1), *NbML1*-allele (Niben101Scf01158g03010.1).

## Results

### Expression and cellular analysis of *Nbwo* and *NbCycB2*

We obtained 14 protein sequences similar to Nbwo and eight similar to NbCycB2 from the NCBI databases. Phylogenetic analyses showed that Nbwo and NbCycB2 had the highest similarity with Slwo and SlCycB2, respectively (Supplementary Fig. S2a, b). One allele of *Nbwo* was identified via BLAST with 95.82% identity and one allele of *NbCycB2* was identified with 95.20% identity. We introduced two mutation sites in *Nbwo* (at loci 2084 and 2092) that were identical to *SlWo*^*V*^ and named the mutant as *NbWo*^*V*^. These two point-mutations caused two amino acid substitutions in the woolly motif (Ile to Arg, Asp to Tyr; Supplementary Fig. S2c). Conserved domain analysis indicated that NbCycB2 contained a WD40-like domain in the N-terminus (NbCycB2-WD40, including an EAR-like motif) and a RING-like domain in the C-terminus (NbCycB2-RING) (Supplementary Fig. S2d). However, no conserved domain of B-type cyclin protein was found in the NbCycB2 sequences.

Subcellular examination indicated that both Nbwo and NbWo^V^ were localized in the nucleus, and that NbcycB2 was localized in the nucleus and cytoplasm (Supplementary Fig. S3a). Y2H assays showed that compared to the positive control containing the BD-53 and AD-T vectors, clones of BD-Nbwo and BD-NbWo^V^ with empty AD combinations could grow on QDO/X/A medium (Supplementary Fig. S3b), while AD-Nbwo and AD-NbWo^V^ with empty BD combinations and the negative control containing the BD-Lam and AD-T vectors failed to grow. This suggested that Nbwo and NbWo^V^ had strong auto-activating ability and that the mutations of the woolly motif did not affect the transactivation ability of Nbwo.

Examination of the spatial expression of *NbCycB2* and *Nbwo* indicated that they were expressed at low levels in the roots but at high levels in organs containing trichomes (Supplementary Fig. S4a). Further investigation showed that GUS was only detected in the leaf and stem trichomes of the GFP-GUS transgenic lines driven by the *NbCycB2* promoter (Supplementary Fig. S4b–c). In transgenic lines driven by the *Nbwo* promoter, although GUS was strongly expressed in the basal and venous regions of young leaves, it was also expressed in the trichomes of cotyledons and young leaves. (Supplementary Fig. S4d).

### NbCycB2 negatively regulates trichome initiation

**Fig. 1.**
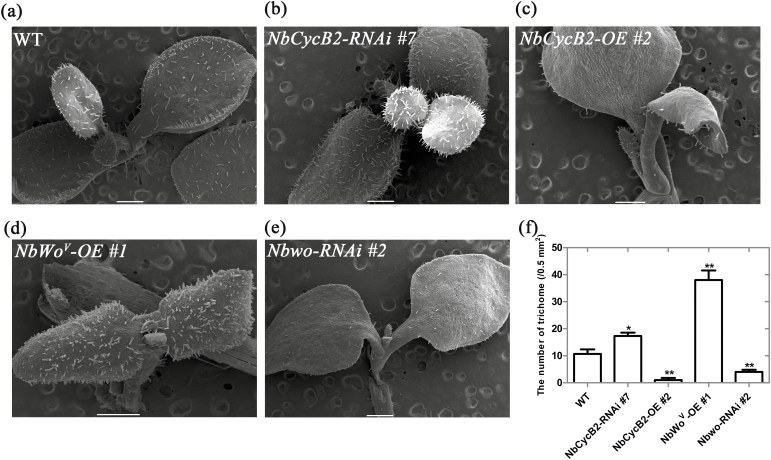
The trichome phenotypes of *N. benthamiana NbCycB2*, *Nbwo*, and *NbWo*^*V*^ transgenic seedlings. SEM images of the trichomes of 10-d-old seedlings of (a) the wild-type (WT), (b) *NbCycB2*-RNAi #7 T_1_, (c) *NbCycB2*-overexpressing (-OE) #2 T_1_, (d) *NbWo*^*V*^-OE #1 T_1_, and (e) *Nbwo*-RNAi #*2* T_1_. Scale bars are 500 μm. (f) The trichome densities of the plants shown in (a–e). Data are means (±SD), *n*=3. Significant differences compared to the wild-type were determined using Student’s *t*-test: *P<0.05, **P<0.01.

Most *NbCycB2*-overexpression (-OE) transgenic T_1_ lines showed a dramatic reduction in the density of trichomes on leaves and stems (Supplementary Fig. S5a, Fig. 1c, f), whilst root length and the number of branch roots significantly increased (Supplementary Fig. S6). Western blotting and qRT-PCR analyses showed that NbCycB2 significantly accumulated in the *NbCycB2-*OE lines, while the expression levels of *Nbwo* and endogenous *NbCycB2* were significantly reduced (Supplementary Fig. S5b, c).

In contrast, the density of trichomes increased significantly on the leaves and stems of 16 *NbCycB2*-RNAi T_1_ lines (Supplementary Fig. S5d, Fig. 1b, f) whilst qRT-PCR analysis showed that the expression level of *NbCycB2* decreased (Supplementary Fig. S5e). This suggested that *NbCycB2* might play a negative role in trichome initiation.

### NbWo^V^ positively regulates trichome initiation

To confirm the function of *Nbwo*, we generated 22 *Nbwo*-knockdown transgenic T_1_ plants (*Nbwo*-RNAi). Compared with the wild-type, the trichome densities were clearly reduced on the leaves and stems of most *Nbwo*-RNAi plants (Supplementary Fig. S7a, e). The efficiency of the RNAi-mediated knockdown was confirmed by qRT-PCR in two independent lines in which the expression of *Nbwo* and *NbCycB2* was significantly reduced (Supplementary Fig. S7b).

**Fig. 2.**
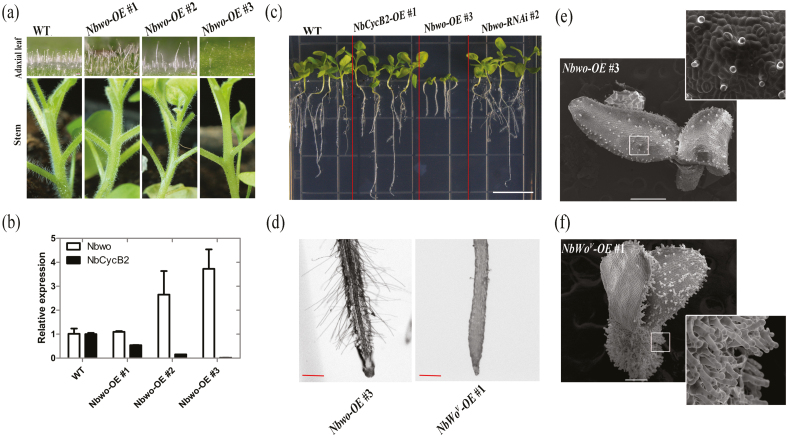
Overexpression of *N. benthamiana Nbwo* causes dwarfism. (a) Phenotypes of *Nbwo*-overexpressing (-OE) transgenic plants. Compared to wild-type (WT) plants, the density of the trichomes in the stems and leaves of the transgenic lines decreased. (b) Relative expression levels of *Nbwo* and *NbCycB2* in the transgenic lines as measured by qRT-PCR. Compared with the wild-type plants, the expression level of *NbCycB2* decreased with the increase in total expression of *Nbwo* in the transgenic plants. Data are means (±SD), *n*=3. Expression is relative to that of the WT, the value of which was set as 1. (c) Root lengths in 2-week-old seedlings of the WT, *NbCycB2*-OE #1 T_1_, *Nbwo*-OE #3 T_1_, and *Nbwo*-RNAi #*2* T_1_. The scale bar is 1 cm. (d) Root hairs of 2-week-old seedlings of *Nbwo*-OE #3 T_1_ and *NbWo*^*V*^-OE #1 T_1_. The root hairs were normal in the *Nbwo*-OE line. The scale bars are 1 mm. (e, f) SEM images of 10-d-old seedlings of (e) *Nbwo*-OE #3 T_1_ and (f) *NbWo*^*V*^-OE #1 T_1_. The trichome density of *Nbwo*-OE plants was normal, whilst the *NbWo*^*V*^-OE plants showed a significant increase. The scale bars are 500 μm. (This figure is available in colour at *JXB* online.)

In common with previous results for tomato (Yang *et al.*, 2011), we found significant increases in the density and branching of trichomes on the leaves and stems of *NbWo*^*V*^*-OE* plants (Fig. 1d, f, Supplementary Fig. S7d, h). qRT-PCR assays showed significantly up-regulated expression levels of *NbWo*^*V*^, endogenous *Nbwo*, and *NbCycB2* in the transgenic lines (Supplementary Fig. S7f, g). Compared to the wild-type, the density of glandular trichomes increased significantly in *NbWo*^*V*^-OE #1, but root hairs were almost absent (Fig. 2d, f). A dwarfism phenotype observed in the T_1_ plants of *NbWo*^*V*^-OE (Supplementary Fig. S8).

### Overexpression of *Nbwo* does not increase trichome density

A total of 20 *Nbwo*-OE plants were generated. Interestingly, the trichome density was found to be negatively related to the expression level of *Nbwo* in T_0_*Nbwo*-OE plants (Fig. 2a, b). The expression levels of *NbCycB2* were significantly reduced in T_0_ plants with reduced trichomes (Fig. 2b). However, the trichome density was decreased in T_1_ plants (Fig. 2e). Compared to the *NbWo*^*V*^-OE #1, the development of trichomes and root hair appeared to be unaffected in the *Nbwo*-OE plants (Fig. 2d-f). These results indicated that the functions of *Nbwo* in trichome and root hair development were quite different to those of *NbWo*^*V*^. However, the higher expression level of exogenous *Nbwo* (e.g. *Nbwo*-OE *#3*) resulted in dwarfism (Fig. 2c), which was similar to the *NbWo*^*V*^-OE lines (Supplementary Fig. S8).

### Nbwo and NbWo^V^ directly target the L1-like box of the *NbCycB2* promoter

**Fig. 3.**
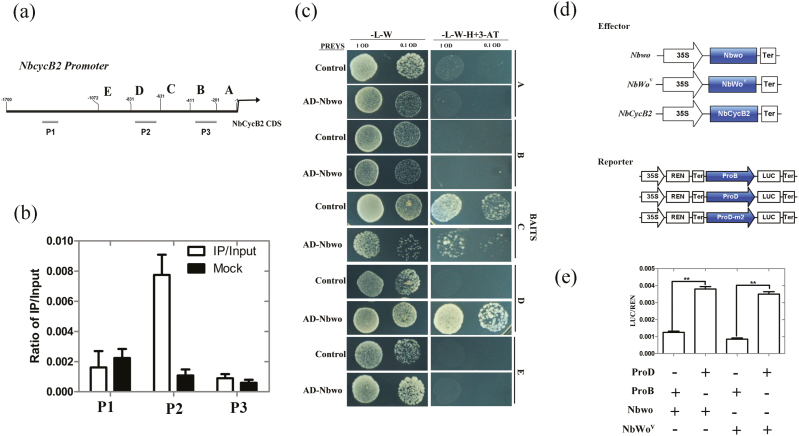
*Nicotiana benthamiana* Nbwo and NbWo^V^ can bind to the *NbCycB2* promoter *in vitro* and *in vivo*. (a) Fragments of the *NbCycB2* promoter were used for ChIP (P1–P3) and yeast one-hybrid (Y1H) assays (A–E). The numbers indicate the positions of the truncations. (b) The ratio of bound promoter fragments versus total input detected by qRT-PCR after immuno-precipitation of HA-NbWo^V^ by HA antibodies. Data are means (±SE), *n*=3. (c) Y1H assays to determine the interactions of the *NbCycB2* promoter fragments and AD-Nbwo or the negative control (AD) in the Y187 yeast strain. (d) Schematic diagram of the effector and reporter constructs used in the LUC assays. (e) Relative reporter activities in *N. benthamiana* protoplasts after transient transformation of the effector and reporter constructs. The relative LUC activity normalized to REN activity are shown (LUC/REN). Data are means (±SD), *n*=3. Significant differences were determined using Student’s *t*-test: **P<0.01. (This figure is available in colour at *JXB* online.)

The expression of *NbCycB2* was significantly up-regulated in the *NbWo*^*V*^-OE lines and decreased in the *Nbwo*-RNAi lines (Supplementary Fig. S7b, f), which indicated that *NbCycB2* was positively regulated by Nbwo and NbWo^V^. To test whether Nbwo directly binds to the promoter of *NbCycB2*, *NbWo*^*V*^-OE plants with *NbWo*^*V*^ fused to the HA tag were analysed using ChIP qRT-PCR assays with a HA antibody. Strong enrichment of NbWo^V^ was observed in the P2 region of the *NbCycB2* promoter in the *NbWo*^*V*^-OE plants (Fig. 3a, b).

To further determine the specific area of binding of Nbwo, we performed Y1H assays. The five truncated fragments of *NbCycB2* promoter that were used are shown in Fig. 3a. Yeast colonies containing the AD-*Nbwo* and *NbCycB2-*proC-pHIS2 or *NbCycB2-*proD-pHIS 2 constructs were grown on selection medium with 60 mM 3-aminotriazole (Fig. 3c). Colonies containing *NbCycB2-*proC-pHIS2 and the empty vector pGADT7 (AD) grew normally on the medium, which indicated that the *NbCycB2* promoter proC fragment possessed high autoactivation activity. This suggested that the D fragment of the *NbCycB2* promoter sequence was the binding targe of the Nbwo protein.

To determine whether Nbwo and NbWo^V^ could directly affect the expression of the D fragment *in vivo*, we performed dual-luciferase assays. The reporters *35S::REN-NbCycB2proD::LUC* and *35S::REN-NbCycB2proB::LUC* and the effectors are shown in Fig. 3d. Each reporter and effector pair was transiently co-expressed in *N. benthamiana* protoplasts (Fig. 3e). Compared to the B fragment, when *Nbwo* or *NbWo*^*V*^ were transiently co-expressed, LUC expression driven by the D fragment was significantly higher in protoplasts. This indicated that the D fragment of the *NbCycB2* promoter appeared to be a specific site for binding of Nbwo and NbWo^V^.

Further analysis of the targeting sequence of *NbCycB2proD* revealed that this sequence contained two L1-like boxes (5´-ATTTACTC-3´) (Supplementary Fig. S9a). When two L1-like boxes were mutated (*proD-m1*, *proD-m2*), Y1H and *in vivo* LUC assays showed that the interaction with the Nbwo protein was abolished (Supplementary Fig. S9b, c). Based on these results, we inferred that the L1-like boxes might be the binding targets of the Nbwo and NbWo^V^ proteins.

### NbCycB2 represses the activity of Nbwo rather than NbWo^V^

**Fig. 4.**
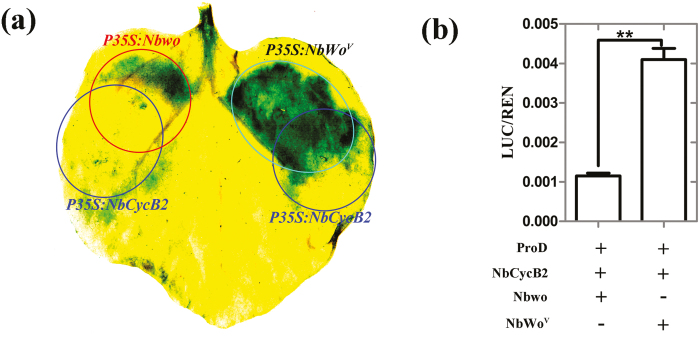
*Nicotiana benthamiana* NbCycB2 can suppress the function of Nbwo, but has no effect on NbWo^V^. (a) Co-expression of *NbCycB2* in leaves of *proNbCycB2-GFP-GUS* transgenic plants inhibits the expression of GUS induced by overexpression of *Nbwo*, but it has no effect on the expression of GUS induced by *NbWo*^*V*^. All the constructs were expressed under the control of the 35S promoter. The areas injected with *P35S::Nbwo*, *P35S::NbWo*^*V*^, and *P35S::NbCycB2* GV3101 strain are indicated. At 72 h after injection, the leaves were stained with GUS substrate. (b) Dual-LUC activity test, confirming that co-expression with *NbCycB2* decreased the transactivation activity of Nbwo compared with NbWo^V^. Data are means (±SD), *n*=3. Significant differences were determined using Student’s *t*-test: **P<0.01.

Since the *NbCycB2*-OE and *Nbwo*-RNAi transgenic lines shared the non-trichome phenotype and the expression of *Nbwo* did not increase in the *NbCycB2*-RNAi lines (Supplementary Fig. S5e), we suspected that NbCycB2 could affect the transactivation ability of Nbwo at the protein levels. To test this hypothesis, *Nbwo*, *NbWo*^*V*^, and *NbCycB2* were transiently co-overexpressed under the control of the 35S promoter in the leaves of the *proNbCycB2::GFP-GUS* transgenic line using *Agrobacterium*-mediated transformation (Fig. 4a). We found that co-expression of *NbCycB2* could inhibit the expression of GUS induced by overexpression of *Nbwo* in the leaves of *proNbCycB2::GFP-GUS* plants. However, GUS expression induced by overexpression of *NbWo*^V^ was not affected by *NbCycB2* expression. These results were further supported by LUC assays, in which co-expression with *NbCycB2* clearly repressed the activity of Nbwo, but did not affect NbWo^V^ (Fig. 4b). These results suggested that NbCycB2 may act as a negative regulator of Nbwo rather than NbWo^V^.

### The interaction between NbCycB2 and Nbwo can be reduced by mutations in the woolly motif

An interaction between SlCycB2 and Slwo has been reported previously (Yang *et al.*, 2011). To explore the domain involved in the physical interaction between NbCycB2 and Nbwo, four truncated fragments of Nbwo containing the HD, LZ domain, START domain, and SAD were used (Supplementary Fig. S10a). Y2H assays suggested that the LZ domain of Nbwo interacted with NbCycB2 (Supplementary Fig. S10b). Bimolecular fluorescence complementation (BiFC) assays were further used to verify the interaction between the Nbwo LZ domain and NbCycB2 *in vivo* (Supplementary Fig. S10c).

**Fig. 5.**
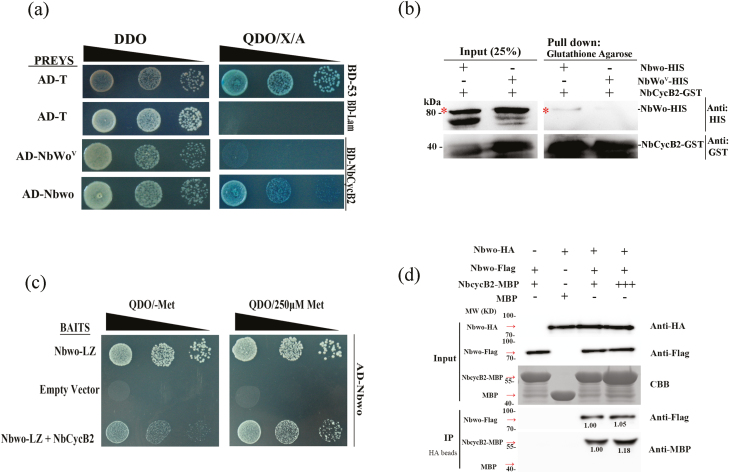
Mutation of the woolly motif attenuates the interaction between Nbwo and NbCycB2 in *N. benthamiana*. (a) Yeast two-hybrid (Y2H) assays of the interactions between NbCycB2 and Nbwo or NbWo^V^ using QDO/X/A medium. Clones with pGADT7-53 (BD-53) and pGADT7-T (AD-T) served as positive controls, and clones with pGBKT7-Lam (BD-Lam) and pGADT7-T (AD-T) served as negative controls. Only the positive control and AD-Nbwo and BD-NbcycB2 could grow on the medium. (b) Pull-down assays between Nbwo or NbWo^V^ with NbCycB2 proteins. The NbCycB2-GST protein was immunoprecipitated with glutathione agarose, and the immunoblots were probed with anti-HIS and anti-GST antibodies. Only the recombinant HIS-Nbwo protein could co-precipitate with GST-NbCycB2. The asterisks indicate the bands for Nbwo or NbWo^V^. (c) Y3H assays to determine the competition between NbCycB2 and the LZ domain of Nbwo for binding to Nbwo (fused to GAL4 DNA-AD). The methionine-repressible promoter in the pBridge vector controlled the expression of *NbCycB2* in the presence of the Nbwo LZ domain (fused to GAL4 DNA-BD). (d) Pull-down assays to determine whether NbCycB2 could compete for binding to Nbwo. The total protein from *P35S::HA-Nbwo* protoplasts was immunoprecipitated with anti-HA beads, and the immunoblots were probed with anti-Flag and anti-MBP antibodies. (This figure is available in colour at *JXB* online.)

Additional Y2H assays were performed to determine whether NbCycB2 also interacted with NbWo^V^. Only the clones of BD-NbCycB2 with AD-Nbwo combinations could grow on the selection medium, while BD-NbCycB2 with the AD-NbWo^V^ combination and the negative control failed to grow (Fig. 5a). These results indicated that NbCycB2 physically interacted with Nbwo but not with NbWo^V^. We then used pull-down and BiFC assays to confirm the lack of interaction between NbCycB2 and NbWo^V^. Purified Nbwo-HIS and NbWo^V^-HIS were incubated with equal amounts of NbCycB2-GST, and immunoblotting showed that only Nbwo-HIS could retain NbCycB2-GST, whereas NbWo^V^-HIS could not (Fig. 5b). For the BiFC analysis, Nbwo and NbWo^V^ were individually fused to the C-terminal part of yellow fluorescent protein (YFP^C^) to generate YFP^C^-Nbwo and YFP^C^-NbWo^V^, while NbCycB2 was ligated to the N-terminal fragment of YFP (YFP^N^) to generate YFP^N^-NbCycB2. We found that co-expression of YFP^C^-Nbwo with YFP^N^-NbCycB2 in *N. benthamiana* protoplasts resulted in strong YFP fluorescence in the nucleus, whereas no YFP signal was observed in the combinations of YFP^C^-NbWo^V^ and YFP^N^-NbCycB2, or in the negative controls (Supplementary Fig. S10e). These results suggested that the interaction between NbCycB2 and Nbwo could be removed by mutations in the woolly motif.

### NbCycB2 does not competitively bind to Nbwo homodimers

It is known that HD-Zip proteins bind to DNA as dimers via the LZ domain (Ariel *et al.*, 2007). To determine whether Nbwo could be dimerized via the LZ domain, we first used Y2H assays to demonstrate that the LZ domain could bind to the Nbwo protein (Supplementary Fig. S10f). BiFC assays were also used to confirm the interaction between the LZ domain and Nbwo (or NbWo^V^) proteins *in vivo* (Supplementary Fig. S10c).

Y3H assays were used to determine whether NbCycB2 could competitively bind to the LZ domain of Nbwo homodimers. The methionine-repressible promoter in the pBridge vector was used to control the expression of *NbCycB2* in the presence of the Nbwo LZ domain (Nbwo LZ domain fused to GAL4 DNA-BD, Nbwo-LZ+NbCycB2), and the pBridge vector containing only the Nbwo LZ domain served as a positive control (Nbwo LZ domain fused to GAL4 DNA-BD, Nbwo-LZ). The methionine promoter is inactive on media with high concentrations of methionine. As shown in Fig. 5c, the clone containing Nbwo-LZ+NbCycB2 was able to grow normally in comparison with the positive control, whether in medium containing methionine (250 μM) or lacking methionine. This suggested that NbCycB2 might not competitively bind to the LZ domain of Nbwo homodimers.

To verify this, we further performed a pull-down assay. Nbwo-HA and Nbwo-Flag proteins were extracted after expression in *N. benthamiana* protoplasts. The total proteins were then uniformly mixed and incubated with the purified NbCycB2-MBP protein. Immunoblot analysis showed that the increased NbCycB2-MBP protein did not affect the amount of Nbwo-Flag retained by Nbwo-HA (Fig. 5d).

### Nbwo can bind to its own genomic DNA

**Fig. 6.**
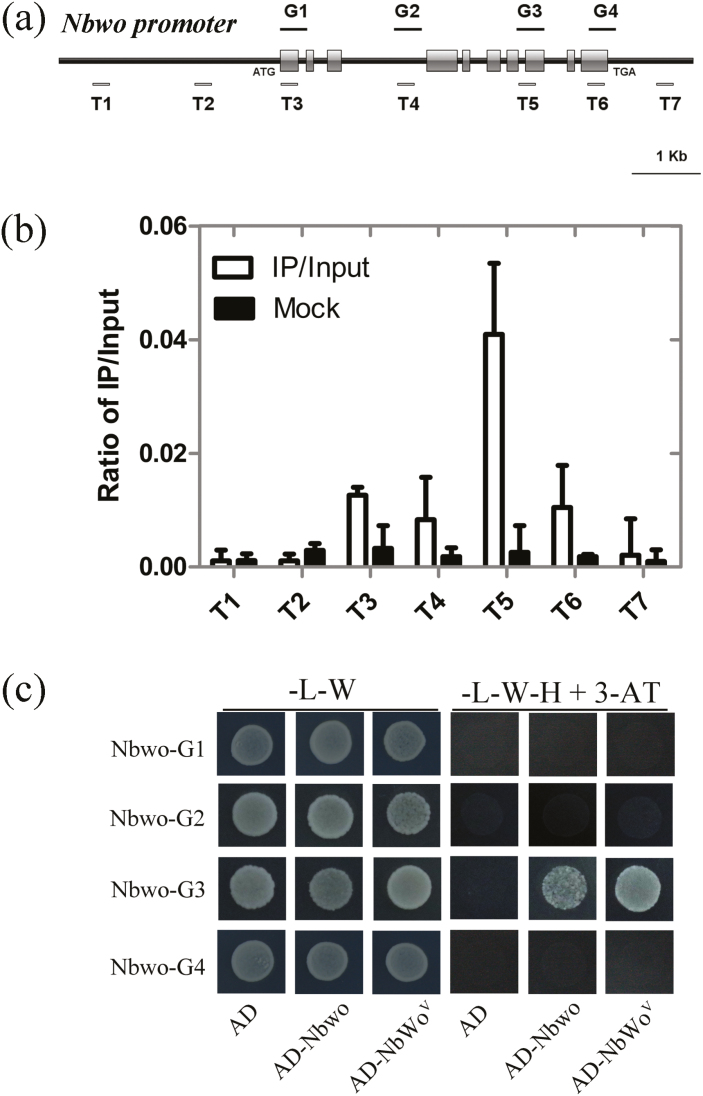
*Nicotiana benthamiana* Nbwo and NbWo^V^ can bind to their own genomic DNA sequences. (a) The fragments of the *Nbwo* genomic sequences used in the ChIP (T1–T7) and yeast one-hybrid (Y1H) assays (G1–G4). (b) Ratio of bound genomic fragments versus total input detected by real-time PCR after immuno-precipitation from the *NbWo*^*V*^-overexpressing (-OE) lines by HA antibodies. Data means (±SE), *n*=3. (c) Y1H assays to determine the interaction of *Nbwo* genomic sequence fragment bait constructs and AD-Nbwo, AD-NbWo^V^, or empty-vector pGADT7 constructs in the Y187 yeast strain. The clones were grown on SD/–Leu/–His/–Trp (–L–W–H) with 60 mM 3-AT medium. (This figure is available in colour at *JXB* online.)

The endogenous expression level of *Nbwo* was reduced in the *NbCycB2*-OE lines (Supplementary Fig. S5b) and increased in the *NbWo*^*V*^-OE plants (Supplementary Fig. S7f), which indicated that Nbwo might be able to regulate its self-expression. To test this hypothesis, ChIP assays were carried out to check whether Nbwo could bind to its genomic DNA sequence in the leaves of the *NbWo*^*V*^-OE transgenic line. Interestingly, an enrichment of NbWo^V^ was detected in the T5 fragment in *NbWo*^*V*^-OE plants (Fig. 6a, b). This result was further demonstrated by Y1H assays, in which only the clones with AD-Nbwo (or AD-NbWo^V^) and Nbwo-G3-pHIS 2 constructs could grow on the resistant medium (Fig. 6c). This suggested that Nbwo and NbWo^V^ could bind to the G3 fragments (including the T5 fragment, Fig. 6a) of its own genomic DNA sequence.

### Overexpression of *NbCycB2* can reduce the dwarf phenotype of *Nbwo*-OE plants

**Fig. 7.**
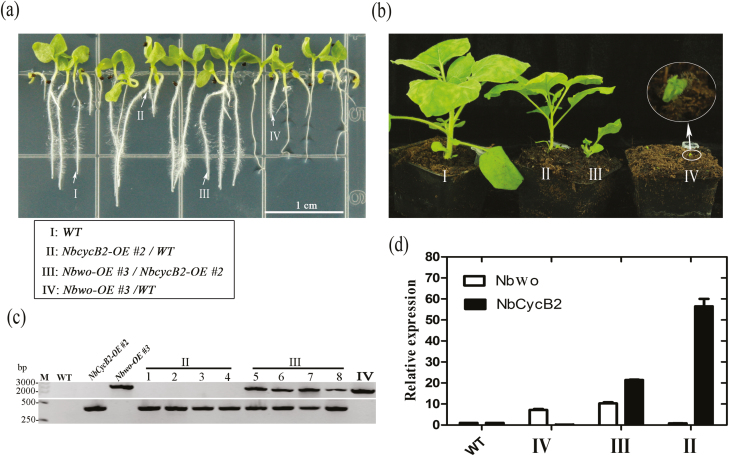
Hybridization between *Nbwo*-overexpressing (-OE) and *NbCycB2*-OE plants of *N. benthamiana*. (a, b) Phenotypes of *NbCycB2*-OE #2 and *Nbwo*-OE #3 hybridization F_1_ plants at (a) 2-weeks-old and (b) at maturity. I, wild-type (WT); II, *NbCycB2*-OE/WT hybrid T_1_; III, *NbCycB2*-OE *#2* and *Nbwo*-OE *#3* hybridization F_1_; IV, *Nbwo*-OE *#3/WT* hybrid T_1_. (c) F_1_ plants were examined using PCR. The reverse primer for the overexpression vector served as the 3´-terminal detection primer, and the forward primer for the detected genes served as the 5´-terminal primer. The WT served as a negative control, and *Nbwo*-OE #3 and *NbCycB2*-OE #2 served as positive controls. No DNA bands were detected in the WT. A ~330-bp DNA band was detected in the *NbCycB2*-OE #2 lines and a ~2199-bp band was detected in the *Nbwo*-OE *#3* lines. In contrast, two bands were detected in *NbCycB2*-OE *#2* and *Nbwo-OE #3* hybridization F_1_ plants. (d) Relative expression levels of *Nbwo* and *NbCycB2* in F_1_ plants as determined by qRT-PCR. Expression is relative to that of the WT, the value of which was set as 1. Data are means (±SD), *n*=3. (This figure is available in colour at *JXB* online.)

To determine whether *NbCycB2* could inhibit the activity of *Nbwo in vivo*, we crossed *NbCycB2-OE #2* T_1_ with *Nbwo-OE #3* T_0_ plants. We found that the dwarf and short-root phenotypes of *Nbwo-OE #3* were indeed reduced by *NbCycB2-OE #2* (Fig. 7a, b). The crossed F_1_ plants were tested using PCR (Fig. 7c), and the expression of *NbCycB2* and *Nbwo* was also verified by qRT-PCR. Compared with T_1_*Nbwo-OE* #3 plants, the balance between *NbCycB2* and *Nbwo* expression was restored in the *NbCycB2-OE* #2 × *Nbwo-OE* #3 crossed F_1_ plants (Fig. 7d).

## Discussion

Trichomes play important roles in plants. They participate in resistance mechanisms to a variety of abiotic stresses such as UV radiation and dehydration (Mauricio and Rausher, 1997; Werker, 2000), and biotic stresses such as pathogenic bacteria and insect herbivores (Freeman and Beattie, 2008; Tian *et al.*, 2012). In tobacco, glandular trichomes function as synthesis and secretion sites of sucrose and diterpenoids (Tissier, 2012), so they are important targets of biotechnological engineering for quality improvement of varieties (Glas *et al.*, 2012). However, few studies so far have examined the development of tobacco trichomes.

*Slwo* is a key gene in tomato and functions to regulate the initiation of multicellular trichomes, with multiple gain-of-function mutant alleles that are capable of increasing the density of trichomes (Among these alleles, the mutation sites of *SlWo* and *SlWo*^*V*^ are both within the woolly motif, Yang *et al.*, 2011). Of these mutants, *SlWo*^*V*^ shows one of the most obvious increases in density (Yang *et al.*, 2015); however, little is known about the mechanisms involved. Expression of *SlCycB2* is significantly increased in *SlWo* and *SlWo*^*V*^ lines, but it is significantly decreased in *Slwo*-RNAi plants (Yang *et al.*, 2011). Because *SlCycB2* is similar to AT5G06270.1, a hypothetical B-type cyclin (now renamed as *AtGIR1*) in Arabidopsis, it was previously thought to have a similar function to that in Arabidopsis, where it promotes the differentiation of unicellular to multicellular trichomes (Schnittger *et al.*, 2005). *SlCycB2* was considered to promote the development of type I trichomes in tomato (Yang *et al.*, 2011); however, the detailed mechanism has not been fully studied.

In our current study, we cloned the homologues of *SlCycB2* and *Slwo* in *N. benthamiana* (named *Nbwo* and *NbCycB2*), and constructed a mutant *Nbwo* allele, *NbWo*^*V*^. We found that overexpression of *NbCycB2* lead to suppression rather than promotion of trichome development on stems and leaves (Fig.1b, Supplementary Fig. S5a). Consistent with qRT-PCR results (Supplementary Fig. S4a), GUS staining assays indicated that *NbCycB2* was specifically expressed in the trichomes of leaves and stems (Supplementary Fig. S4b, c), and that *Nbwo* was also expressed in the trichomes of cotyledons and young leaves (Supplementary Fig. S4d). These results suggested that NbCycB2 may serves as a negative regulator of trichome initiation. None of the B-type cyclin conserved domains were found in the SlCycB2 and NbCycB2 protein sequences (Supplementary Fig. S2d). Hence, whether SlCycB2 can function as a B-type cyclin protein requires further study.

### NbWo^V^ and Nbwo directly regulate the expression of *NbCycB2* through binding to the L1-like boxes in its promoter

*SlCycB2* has been reported to be indirectly regulated by Slwo (Yang *et al.*, 2011, 2015). However, we found that the expression level of *NbCycB2* was up-regulated in *NbWo*^*V*^-OE plants and down-regulated in *Nbwo*-RNAi lines (Supplementary Fig. S7), indicating that *NbCycB2* might be the downstream gene of *Nbwo*. ChIP, Y1H, and LUC assays confirmed that the D fragments of the *NbCycB2* promoter were the binding target of Nbwo and NbWo^V^ (Fig. 3). Mutation of the two L1-like box sequences in *NbCycB2*proD inhibited the binding of Nbwo and NbWo^V^ both *in vitro* and *in vivo* (Supplementary Fig. S9 a–c). We thus demonstrated that the expression of *NbCycB2* was directly regulated by Nbwo and NbWo^V^ through the binding of L1-like boxes in the promoter. Using ChIP and YIH assays, we further demonstrated that Nbwo and NbWo^V^ could self-regulate their own endogenous expression by binding to their own genomic DNA sequences (Fig. 6).

### Increased trichome density is induced by mutation of the *Nbwo* woolly motif

*NbWo* ^*V*^ contained only two point-mutations (at loci 2084 and 2092 of the *Nbwo* CDS), which caused two amino acid replacements in the woolly motif of the Nbwo protein (Ile to Arg, Asp to Tyr; Supplementary Fig. S2c). However, these mutations caused a large difference between the functioning of Nbwo and NbWo^V^. Although overexpression of either *Nbwo* or *NbWo*^*V*^ could cause a dwarf phenotype in offspring (Fig. 2c, Supplementary Fig. S6a), the trichome phenotypes of were completely different. Trichome density was decreased with the expression of *Nbwo* in the T_0_ generation of the *Nbwo*-OE lines (Fig.2a, b). In contrast, as *NbWo*^*V*^ expression increased in the *NbWo*^*V*^-OE lines, the density and branching of the trichomes on the leaves were also significantly increased (Fig. 2f, Supplementary Fig. S7d, f). These results implied that the *Nbwo* SAD (including the woolly motif) might itself possess repression activity. However, compared to wild-type, there was no difference in trichome density in the *Nbwo*-OE lines where the SAD had been deleted (Supplementary Fig. S11). This suggested that deletion of the SAD might disrupt the function of *Nbwo*, and that mutation of the woolly motif in the SAD was important for enhancing the functioning of *Nbwo*.

### NbCycB2 represses the transactivation activity of Nbwo at the protein level

Our results demonstrated that *NbCycB2* was directly regulated by Nbwo; however, similar non-trichome phenotypes were found in the *NbCycB2*-OE and *Nbwo*-RNAi transgenic lines (Supplementary Figs S5a, S7a). In addition, the expression of *Nbwo* was not increased in *NbCycB2*-RNAi plants Supplementary (Fig. S5e). This suggested that NbCycB2 might repress the transactivation activity of Nbwo at the protein level. GUS expression was up-regulated by the expression of *Nbwo* and inhibited by the co-expression of *NbCycB2* in the leaves of the *NbCycB2pro::GFP-GUS* transgenic line (Fig. 4a), and the same result was found in LUC assays (Fig. 4b). In addition, hybridization with *NbCycB2*-OE was able to attenuate the dwarf phenotype of T_1_*Nbwo*-OE (Fig. 7a, b). Further examination showed that the expression levels of endogenous *NbCycB2* and *Nbwo* were reduced in the *NbCycB2*-OE lines (Supplementary Fig. S5b), and they were found to be downstream of the regulatory genes of Nbwo (Figs 3, 6). Taken together, these results supported our hypotheses that NbCycB2 may act as a negative regulator of Nbwo at the protein level.

In a previous study, SlCycB2 was reported to interact with Slwo (Yang *et al.*, 2011). Further investigation of the interaction between Nbwo and NbCycB2 revealed that the dimerized LZ domain of Nbwo binds to NbCycB2 (Supplementary Fig. S10b, c). Using Y2H, BiFC, Y3H, and co-IP assays we also found that Nbwo could form a homodimer through the LZ domain, and the NbCycB2 protein did not competitively bind to the LZ domain of Nbwo (Fig. 5c, d, Supplementary S10b, c). These results indicated that NbCycB2 might bind to the Nbwo protein via its LZ domain to repress its transactivation ability by recruiting another inhibitor. However, further study is required to determine whether *NbCycB2* functions similarly to its Arabidopsis homologues *AtGIR1* and *AtGIR2*, which also interact with the co-repressor TOPLESS (Long *et al.*, 2006, Szemenyei *et al.*, 2008, Pauwels *et al.*, 2010, Wu and Citovsky, 2017*b*).

### The interaction between Nbwo and NbCycB2 is blocked by mutation in the Nbwo woolly motif

In Arabidopsis, feedback-loop regulation mechanisms of R3 MYBs (TRY, CPC, and others) occur through competitively binding to GL3/EGL3 to form a non-functional trimeric protein complex (MYB-bHLH-WDR) that inhibits the formation of trichomes (Wang *et al.*, 2008, Wester *et al.*, 2009). Feedback-loop regulation has been reported as an effective strategy for many HD-ZIP proteins to maintain normal organism development (Ohgishi *et al.*, 2001, Williams and Fletcher, 2005, Kim *et al.*, 2008, San-Bento *et al.*, 2014). However, trichome formation was not repressed by the high expression level of *NbCycB2* in *NbWo*^V^*-*OE plants (Supplementary Fig. S7d, f), which suggested that the negative effect of *NbCycB2* could be eliminated by the mutation in *NbWo*^*V*^. This hypothesis was verified by LUC and GUS activity assays, which showed that the transactivation activity of *NbWo*^*V*^ was not affected by the expression or non-expression of *NbCycB2* (Fig. 4a, b).

Further investigation determined that the interaction between NbCycB2 and Nbwo could be blocked by mutation of the woolly motif in the NbWo^V^ protein both *in vitro* and *in vivo* (Fig. 5a, b, Supplementary S10d). Elimination of the interaction prevented NbWo^V^ from being inhibited by NbCycB2. The high expression levels of *NbCycB2* and endogenous *Nbwo* in the *NbWo*^*V*^-OE lines further supported this hypothesis (Supplementary Fig. S7f).

### Conclusions

In summary, we found that NbCycB2 was specifically expressed in the trichomes of *N. benthamiana* and negatively affected trichome formation. Further analysis revealed that Nbwo and NbWo^V^ directly regulated the expressions of *NbCycB2* and *Nbwo* by binding to the L1-like box in the *NbCycB2* promoter and its own genomic DNA sequence. In addition, NbCycB2 may function via binding to the LZ domain of Nbwo, which represses the activity of Nbwo and reduces the expression of genes downstream of *Nbwo* , eventually leading to the inhibition of trichome initiation. The interaction between NbCycB2 and Nbwo could be blocked by a mutation in the woolly motif (the *Nbwo* gain-of-function mutation allele, *NbWo*^*V*^), which prevented repression by NbCycB2 and resulted in a dramatic increase in trichome density and branching. A model of the regulation network is given in Supplementary Fig. S12. Our study has determined the relationship between *NbCycB2* and *Nbwo*, the key regulatory genes of multicellular trichomes in *N. benthamiana*, and hopefully will facilitate biotechnological engineering and research on multicellular trichomes more generally in plants.

## Supplementary data

Supplementary data are available at *JXB* online.

Fig. S1. SEM images of trichomes in the leaves of *N. benthamiana*.

Fig. S2. Sequence analysis of *Nbwo*, *NbCycB2*, and their similar proteins.

Fig. S3. Subcellular localization and auto-activation of *NbCycB2*, *Nbwo*, and *NbWo*^*V*^.

Fig. S4. Expression patterns of *NbCycB2* and *Nbwo* in *N. benthamiana* plants.

Fig. S5. Overexpression of and RNA-interference of *NbCycB2* in *N. benthamiana*.

Fig. S6. Root phenotypes of wild-type, *NbCycB2*-RNAi #7 T_1_, *NbCycB2*-OE #2 T_1_, *NbWo*^*V*^-OE #1 T_1_, and *Nbwo*-RNAi #*2* T_1_ seedlings.

Fig. S7. RNA-interference of *Nbwo* and overexpression of *NbWo*^*V*^ in *N. benthamiana*.

Fig. S8. The phenotypes of *NbWo*^*V*^-OE lines.

Fig. S9. Nbwo and NbWo^V^ bind directly to L1-like boxes of the *NbCycB2* promoter.

Fig. S10. The interactions between Nbwo and NbCycB2, and Nbwo and the Nbwo LZ domain.

Fig. S11. The phenotype of the overexpressing *Nbwo-SAD-mutant* in *N. benthamiana*.

Fig. S12. A simplified model for regulation between *Nbwo* and *NbCycB2*.

Table S1. List of similar proteins for Nbwo.

Table S2. List of similar proteins for NbCycB2.

Table S3. Primers used in this study.

## Supplementary Material

erz542_suppl_Supplementary_Figures

erz542_suppl_Supplementary_Table_1

erz542_suppl_Supplementary_Table_2

erz542_suppl_Supplementary_Data
