# Supplementary material for: NbCycB2 represses Nbwo activity via a negative feedback loop in tobacco trichome development
Source: J Exp Bot. 2020 Jan 28;71(6):1815–27. doi: 10.1093/jxb/erz542 (PMC7242068; doi:10.1093/jxb/erz542)
Supplement: erz542_suppl_Supplementary_Figures [file erz542_suppl_supplementary_figures.pdf]

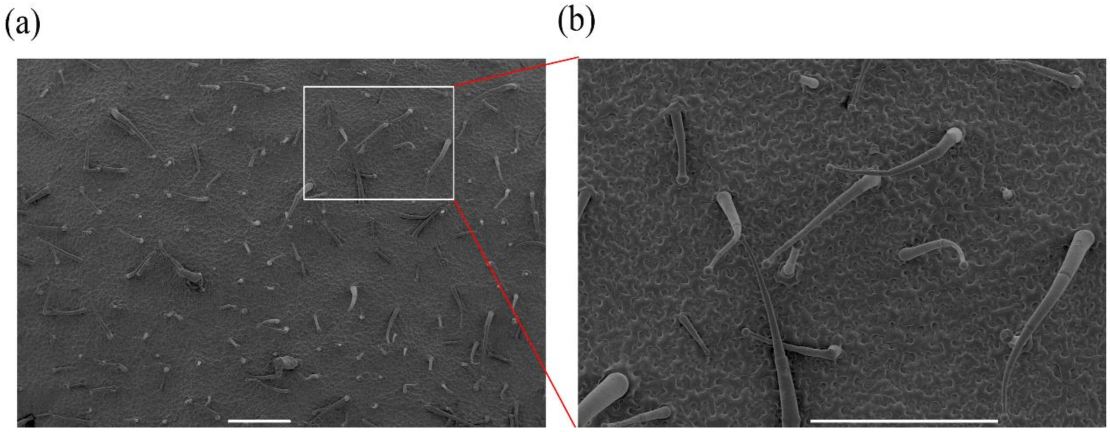


Fig. S1: Scanning electron micrographs (SEMs) of trichomes in the leaf of *N. benthamiana*

(a) The trichome types in the leaf of *N. benthamiana* are shown. (b) Magnification of the white box area in (a). Almost all trichomes in *N. benthamiana* were glandular. The bar is 500 µm.

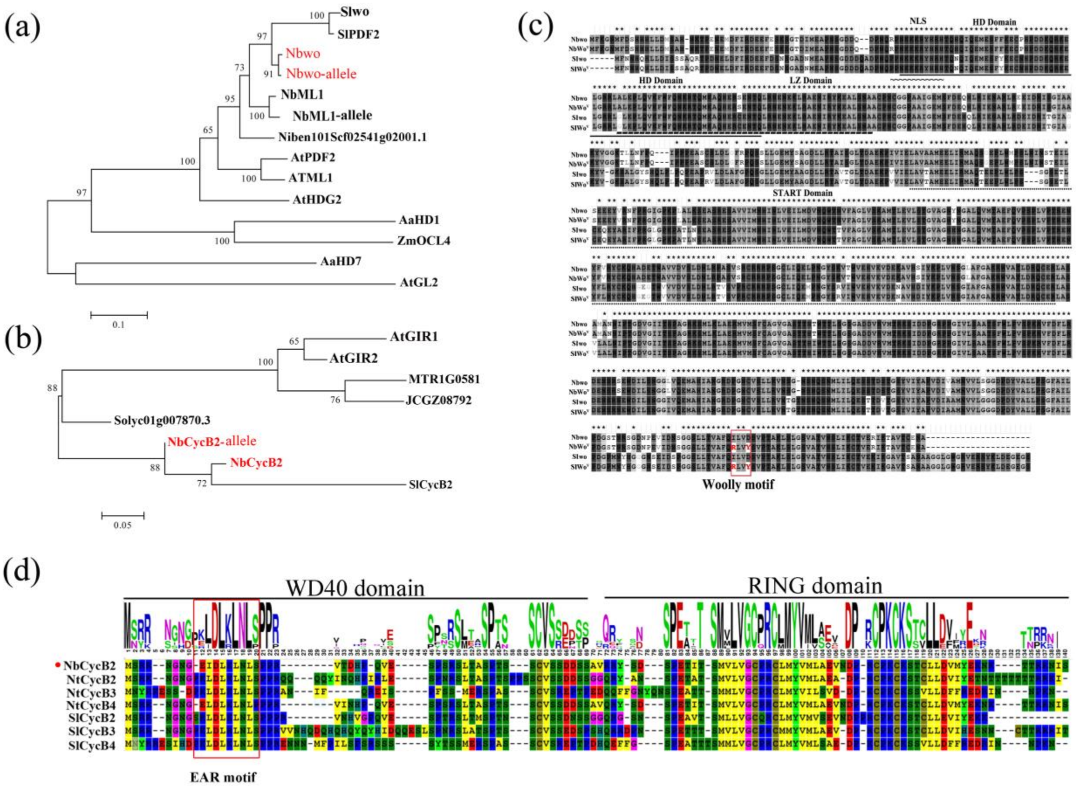


Fig. S2: Sequence analysis of Nbwo, NbCycB2 and their similar proteins

(a) The phylogenetic tree shows the relationships between Nbwo protein and other HD-ZIP IV proteins. Nbwo and its allele were shown in red. (b) The phylogenetic tree shows the relationships between NbCycB2 protein and other similar proteins. NbCycB2 and its allele were shown in red. Protein sequence alignment and phylogenetic tree construction were performed in MEGA 5.2 software using the maximum-likelihood (ML) criterion with 100 bootstrap replicates. (c) Protein sequence alignment between Nbwo, $NbWo^{V}$, Slwo and $SlWo^{V}$. The Nbwo allele ($NbWo^{V}$) carried a mutation at 697 and 700 in the woolly motif [isoleucine (I) to arginine (R) and aspartic acid (D) to tyrosine (Y)]. The red box is the Woolly motif sequence. (d) Analysis of NbCycB2 conserved protein domains. The red box is the EAR motif protein sequence (KLDLKLNL).

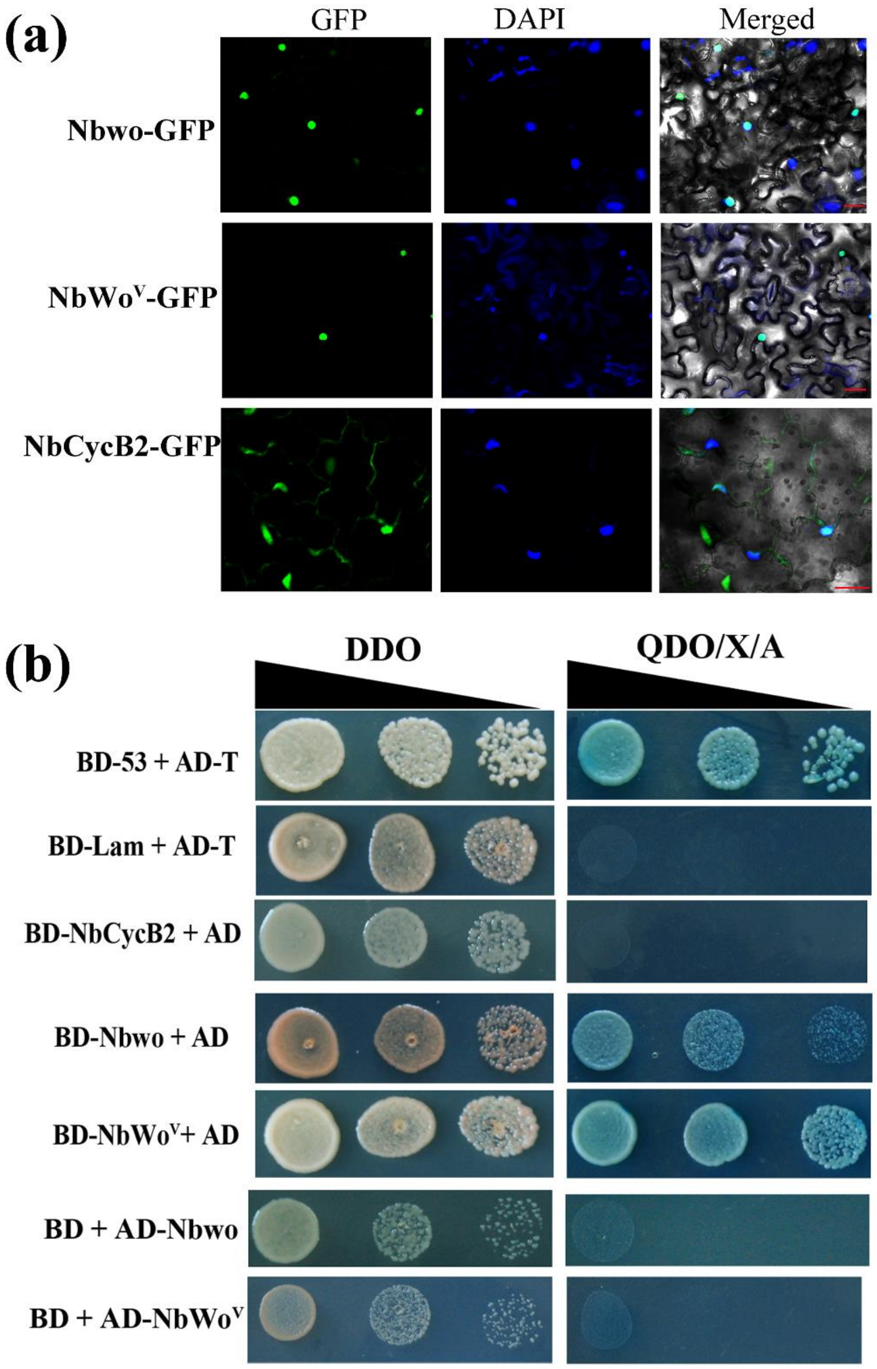


Fig. S3: The subcellular localization assay and auto activation test of *NbCycB2*, *Nbwo* and *NbWo*$^V$

(a) The subcellular localization of *NbCycB2*, *Nbwo* and *NbWo*$^V$ was determined in *N. benthamiana* leaves. As shown in the fluorescence and bright field images, NbCycB2, Nbwo and NbWo$^V$ localized in the nucleus (bars, 50 μm).

(b) The auto activation test of *NbCycB2*, *Nbwo* and *NbWo*$^V$ is shown. *NbCycB2*, *Nbwo* and *NbWo*$^V$ were fused to the GAL4-binding domain in the pGBKT7 vector and the

GAL4-activation domain in the pGADT7 vector and co-transformed with empty pGADT7 or pGBKT7 vector in Y2HGold yeast, respectively. After selection on QDO/X/A medium, blue clones indicated that these baits had auto-activation ability. Clones with pGBKT7-53 (BD-53) and pGADT7-T (AD-T) served as the positive controls, and pGBKT7-Lam (BD-Lam) and pGADT7-T (AD-T) were negative controls.

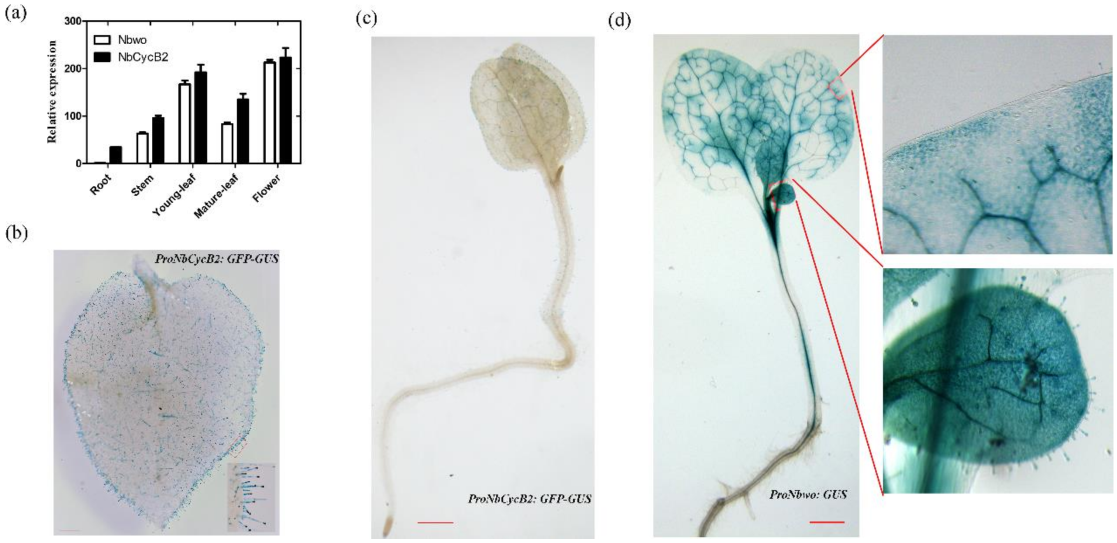


Fig. S4: The expression pattern of *NbCycB2* and *Nbwo* in *N. benthamiana*

(a) The relative expression levels of *NbCycB2* and *Nbwo* respectively were measured by real-time PCR in root, stem, young leaf, mature leaf and flower of *N. benthamiana*. Actin was used as an internal reference, and the expression of *Nbwo* in root served as the control. Data are presented as the means and SD (n = 3). (b), (c) GUS staining was detected in the mature leves and seedlings of *ProNbCycB2*: *GFP-GUS* and *ProNbwo*: *GUS* transgenic lines (bars, 200 µm). (d) GUS staining was detected in the young leves of *ProNbwo*: *GUS* transgenic lines (bars, 200 µm).

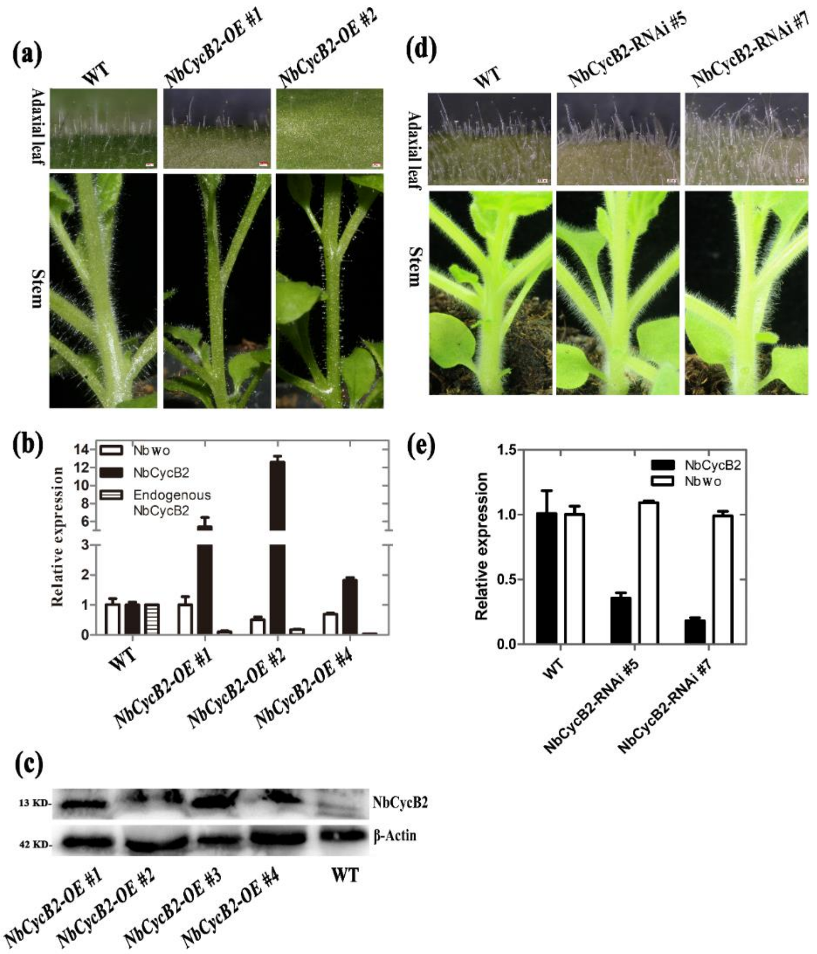


Fig. S5: Overexpression of *NbCycB2* and RNA interference of *NbCycB2* in *N. benthamiana*

(a) The trichome phenotypes on the stem and mature leaves of *NbCycB2-OE* (*P35S: NbCycB2*) transgenic lines was shown. The trichome density was clearly reduced in the *NbCycB2-OE* lines. (b) The expression levels of *NbCycB2* and *Nbwo* were measured by real-time PCR in the *NbCycB2-OE* transgenic lines. Data are presented as the means and SD (n = 3). (c) The Flag-NbCycB2 fused proteins were detected by western blotting in the *NbCycB2-OE* transgenic lines. (d) Trichomes derived from stems and leaves of *NbCycB2-RNAi* transgenic lines. (e) Expression of exogenous *NbCycB2* and *Nbwo* was detected by real-time PCR in the *NbCycB2-RNAi* lines. Error bars represent the SD (n = 3).

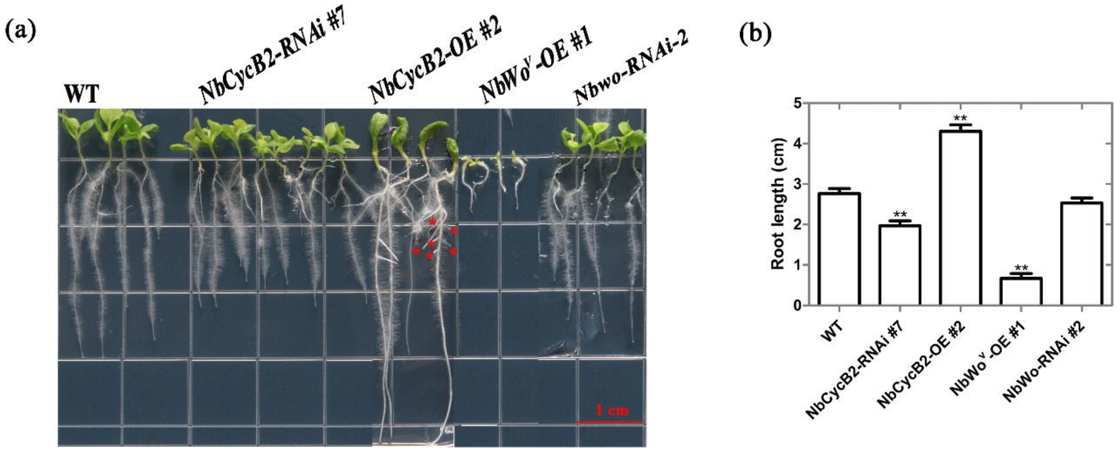


Fig. S6: The root phenotypes of wild type, *NbCycB2-RNAi* #7 T1, *NbCycB2-OE* #2 T1, *NbWo*$^{V}$*-OE* #1 T1, and *Nbwo-RNAi #2* T1 seedlings

(a) The root phenotypes of wild type, *NbCycB2-RNAi* #7 T1, *NbCycB2-OE* #2 T1, *NbWo*$^{V}$*-OE* #1 T1, and *Nbwo-RNAi #2* T1 two-weeks-old seedlings. The red bar is 1 cm.

(b) The root length of wild type, *NbCycB2-RNAi* #7 T1, *NbCycB2-OE* #2 T1, *NbWo*$^{V}$*-OE* #1 T1, and *Nbwo-RNAi #2* T1 two-weeks-old seedlings. "**" Indicates a significant difference at $P < 0.01$ by Student's t test compared with WT. Error bars represent the SD ($n = 3$).

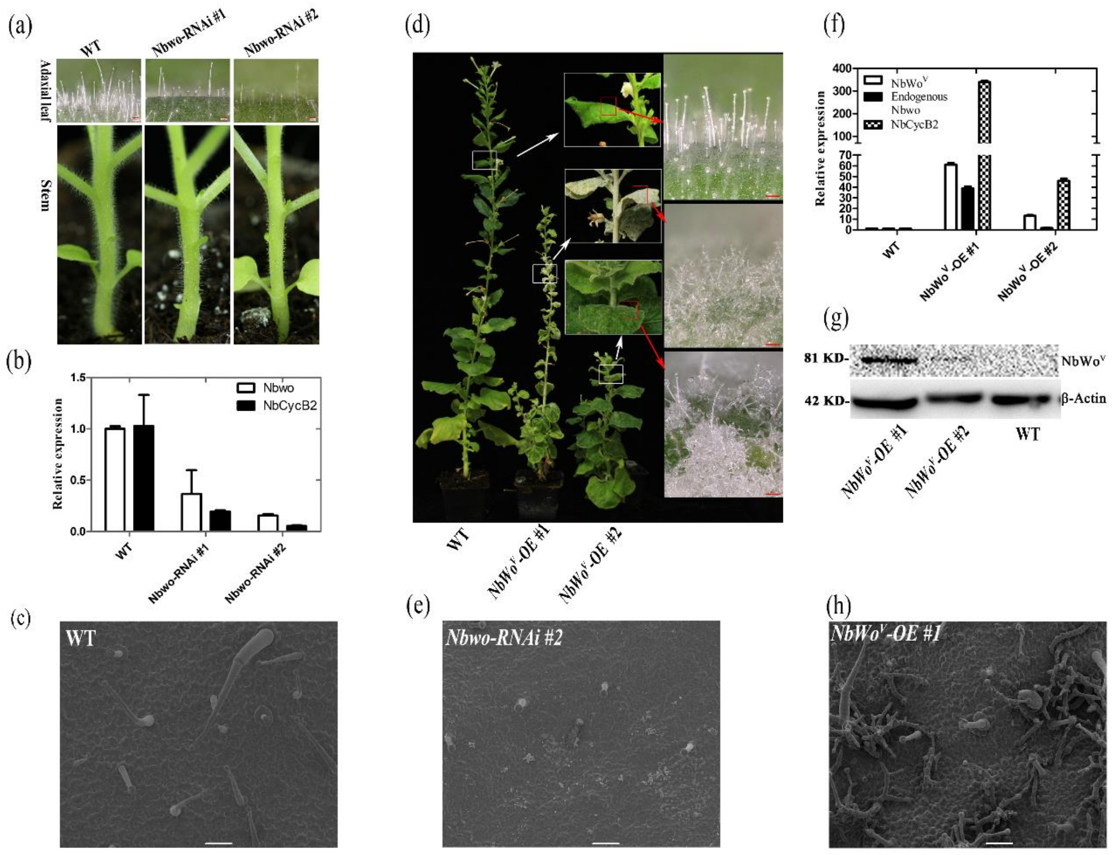


Fig. S7: RNA interference of *Nbwo* and overexpression of *NbWo*$^{V}$ in *N. benthamiana*

(a) Trichomes on stem and leaves derived from the *Nbwo-RNAi* transgenic lines. (b) The expression levels of *NbCycB2* and *Nbwo* were measured by RT-PCR in the *Nbwo-RNAi* transgenic lines. Data are presented as the means and SD (n = 3). RNAi interferes with the expression of *Nbwo*, which significantly reduced the number of trichomes in *N. benthamiana* leaf and stem epidermis. (c), (e), (h) SEMs of trichomes in the mature leaves of wild type, *Nbwo-RNAi #2* and *NbWo*$^{V}$*-OE #1* lines are shown. The white bar is 100 μm. (d) The phenotype of *NbWo*$^{V}$*-OE* lines. Over-expression of *NbWo*$^{V}$ clearly induced an increased trichome density and branching. (f) The expression level of *NbWo*$^{V}$, *NbCycB2* and endogenous *Nbwo* were detected by RT-PCR in the *NbWo*$^{V}$*-OE* lines. Error bars represent the SD (n = 3). (g) HA-NbWo$^{V}$ fusion protein detected by western blotting in *NbWo*$^{V}$*-OE* transgenic lines.

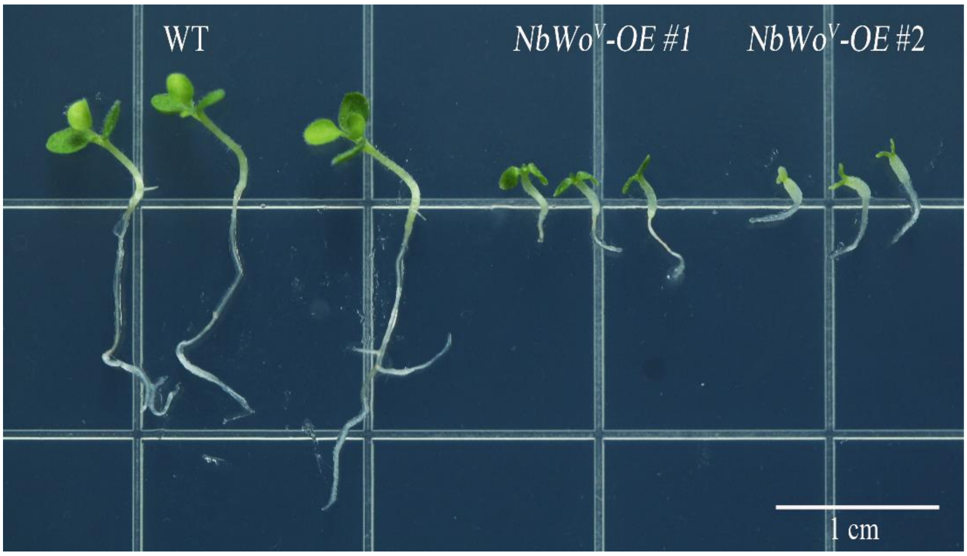


Fig. S8: The phenotype of *NbWo*$^V$*-OE* lines

Compared with WT, *NbWo*$^V$*-OE* #1*,* #2 lines displayed dwarfism (10-day-old seedlings).

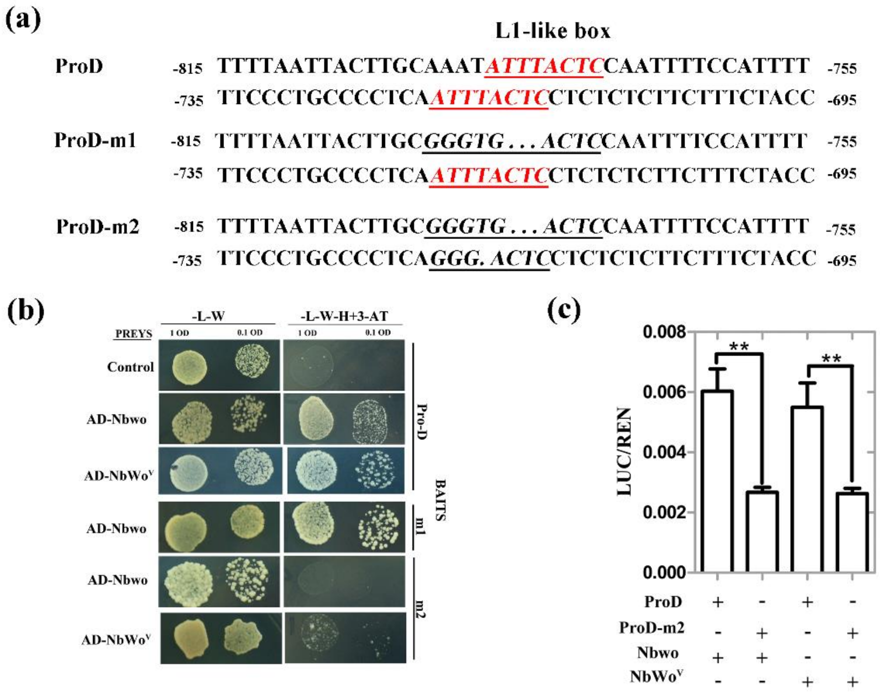


Fig. S9: Nbwo and NbWo$^{V}$ bind directly at the L1-like boxes of the *NbCycB2* promoter (a) The DNA sequences of wild type *NbCycB2* promoter (ProD) and mutant fragments are shown. L1-like boxes (ATTTACTC) are indicated in red italic letters. The mutated bases in the m1 and m2 sequences are underlined. (b) One-hybrid (Y1H) assays were used to detect the interaction between mutant D fragments of *NbCycB2* promoter bait constructs and AD-*Nbwo* or AD-*NbWo$^{V}$* in Y187 yeast strains, and the empty pGADT7 construct served as the control. (c) Relative reporter activity was measured in *N. benthamiana* protoplasts after transient co-transformation of the effector and reporter constructs. The relative LUC activities normalized to REN activity are shown (LUC/REN). “**” Represents a significant difference in the LUC activity between the D and m2 fragment reporter following their co-transformation with *Nbwo* or *NbWo$^{V}$* effector, respectively ($P < 0.01$, Student's t test). Error bars represent the SD ($n = 3$).

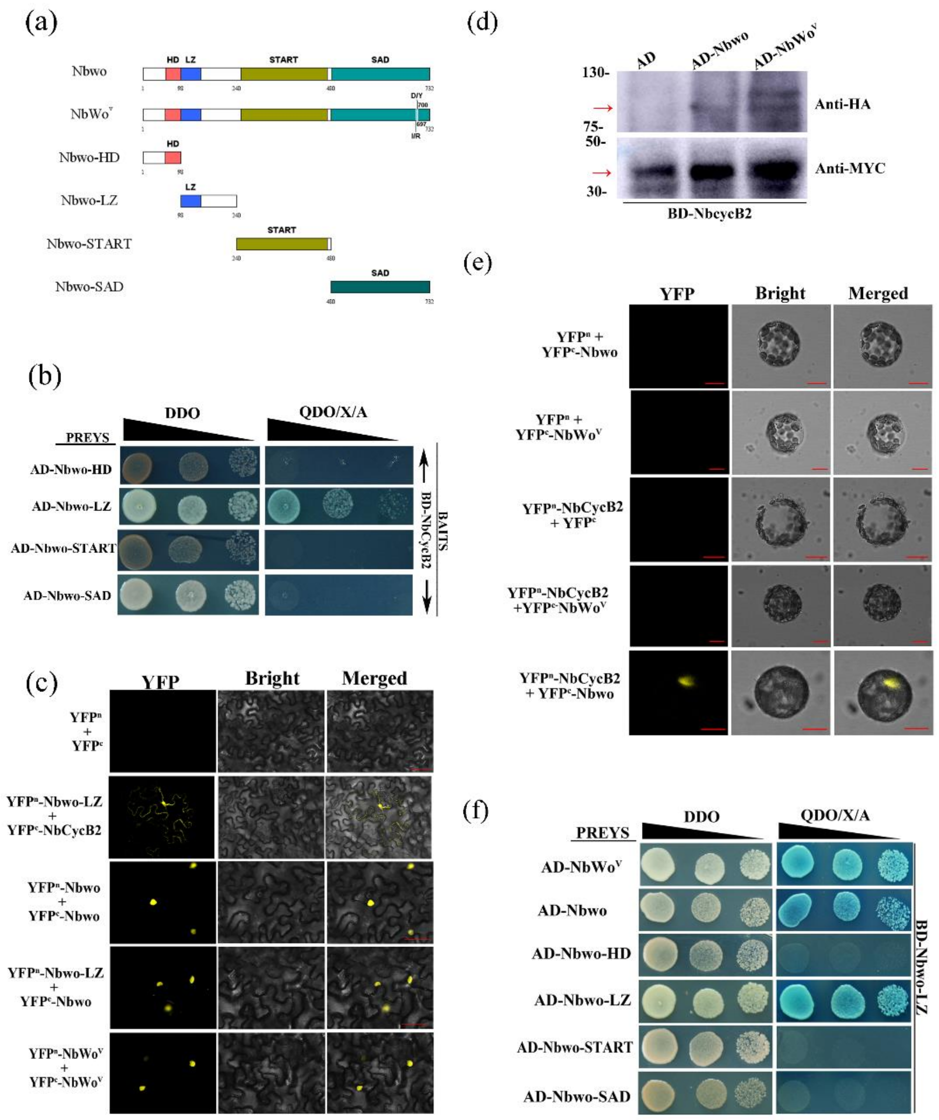


Fig. S10: The interaction between Nbwo and NbCycb2, Nbwo and the Nbwo LZ domain.

(a) Schematic diagrams of Nbwo protein domain constructs. The numbers indicate positions of the first and last amino acid of the Nbwo truncations. (b) The interaction between NbCycB2 and the Nbwo domains protein were determined using the Y2H system. (c) The interaction between Nbwo and NbCycB2 or itself were demonstrated using the BiFC assay. Each indicated pair of constructs was co-infiltrated into leaves of *N. benthamiana* (Bar, 50 μm). (d) The expression levels of Nbwo, NbWo$^{V}$ and NbCycB2 in the yeast strains are shown in Fig. 5a. AD-Nbwo, AD-NbWo$^{V}$ and AD

(empty pGADT7 vector, GAL4 DNA-AD fused to HA tag) were detected with anti-HA antibody (top). The BD-NbcycB2 ptotein (GAL4 DNA-BD fused to MYC tag in the pGBKT7 vector) was detected with anti-MYC antibody (bottom). (e) The interaction between NbCycB2 and Nbwo or NbWo$^{V}$ proteins was determined by the BiFC assay in *N. benthamiana* protoplasts (bars, 20 μm). Yellow fluorescence indicates positive protein-protein interactions. (f) Detection of the interaction between the Nbwo and Nbwo LZ domain using the yeast two-hybrid assay. Blue clones grown on QDO/X/A medium indicated positive protein-protein interactions.

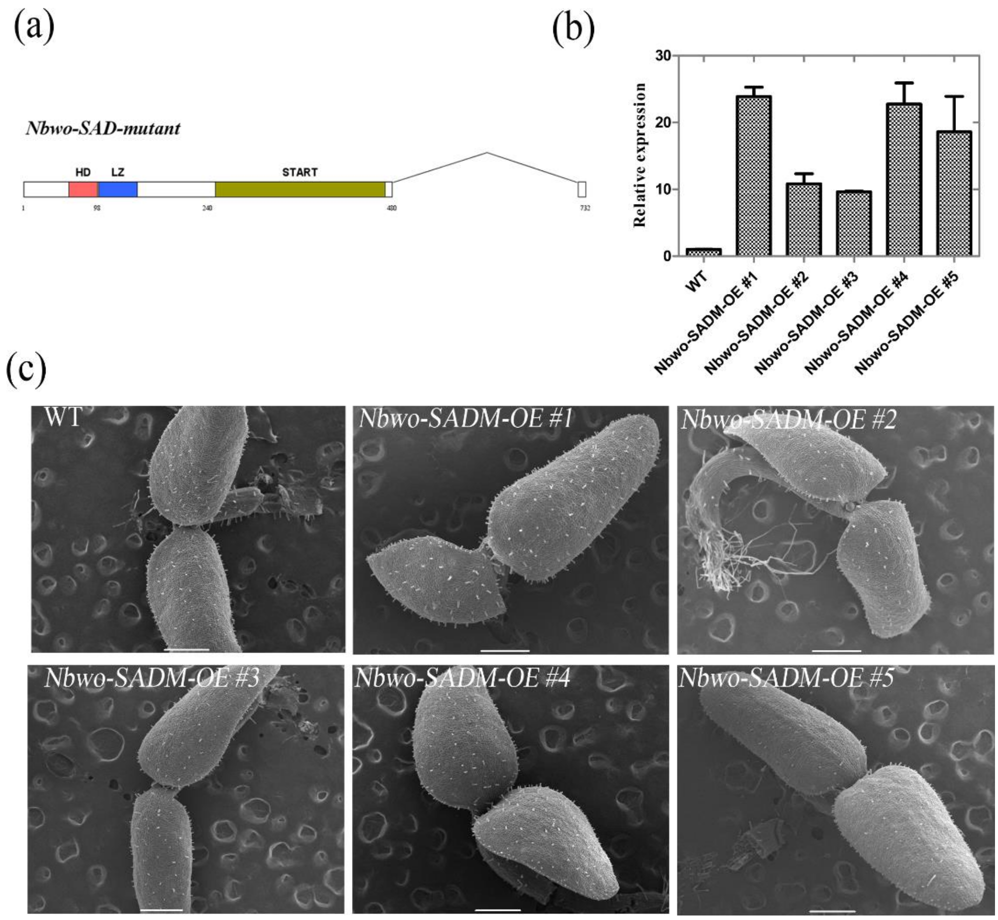


Fig. S11: The phenotype of the overexpressing *Nbwo-SAD-mutant* in *N. benthamiana*. (a) Schematic diagrams of the mutation of the Nbwo protein SAD domain. (b) The relative expression levels of *Nbwo-SAD-mutant* (*Nbwo-SADM*) were measured by qRT-PCR in F1 plants. Data are presented as the means and SD (n = 3). (c) Scanning

electron micrographs (SEMs) of trichomes in the leaf of *Nbwo-SADM* CDS overexpression lines. Compared with wild type, the trichome density showed no significant increase in one-week-old seedlings of the *Nbwo-SADM* overexpression lines (*Nbwo-SADM-OE*). The white bar is 500 µm.

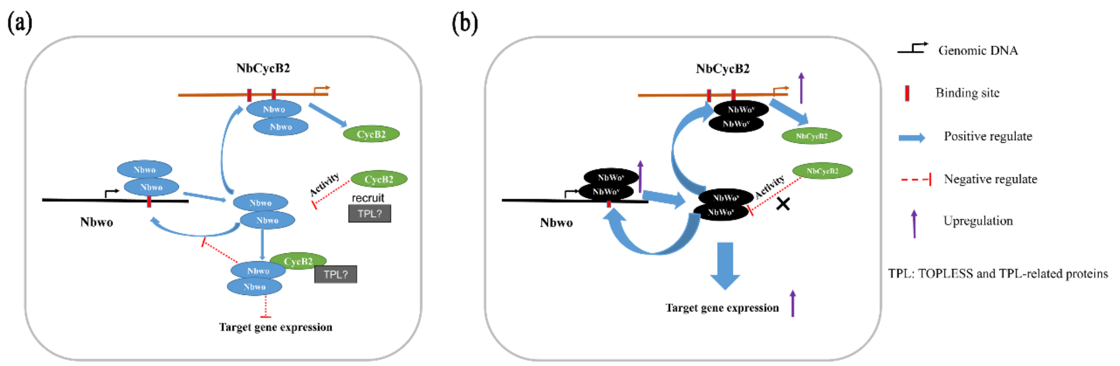


Fig. S12: A simplified model of the regulation between *Nbwo* and *NbCycb2*

(a) Both Nbwo and NbWo$^{V}$ proteins can self-dimerize into homodimers, which then activate downstream genes to promote trichome initiation. Moreover, the homodimers can also promote the expression of *NbCycb2* and self-regulate their endogenous expression through binding to the *NbCycb2* promoter and their own genomic sequences, respectively. In contrast, NbCycb2 binds to the Nbwo dimer and may inhibit trichome initiation by inhibiting the transactivation of Nbwo. However, whether NbCycb2 participates in inhibitory activities through the recruitment of a TOPLESS-like co-repressor requires further study. In summary, *Nbwo* and *NbCycb2* form a negative feedback loop to regulate trichome development. (b) Since NbWo$^{V}$ does not interact with NbCycB2, it is protected from inhibition by NbCycB2 and promotes the expression of its downstream genes, which may results in a significant increase in the density and branching of trichomes in the *NbWo$^{V}$* mutant lines.
